# Preliminary Characterization of Predictive Factors of the Visual Change after Epi-On and Epi-Off Corneal Collagen Crosslinking Techniques

**DOI:** 10.1155/2021/9680253

**Published:** 2021-12-07

**Authors:** Sidi Mohamed Hamida Abdelkader, Joaquín Fernández, Javier Sebastián, David P. Piñero

**Affiliations:** ^1^Department of Ophthalmology, Torrecárdenas Hospital Complex, 04009 Almería, Spain; ^2^Department of Ophthalmology (Qvision), Vithas Virgen del Mar Hospital, 04120 Almeria, Spain; ^3^Department of Ophthalmology, Vithas Medimar International Hospital, 03016 Alicante, Spain; ^4^Department of Optics, Pharmacology, and Anatomy, University of Alicante, Alicante, Spain

## Abstract

**Purpose:**

To investigate the potential predictive factors of the visual change achieved with accelerated epi-on and epi-off corneal collagen crosslinking (CXL) in keratoconus.

**Methods:**

This retrospective comparative study analyzed 67 eyes treated with an accelerated epithelium-on (epi-on group) and epithelium-off (epi-off group) CXL. The clinical outcomes were evaluated and compared during a 1-year follow-up. Likewise, the relationship of the change achieved with both CXL techniques in the corrected distance visual acuity (CDVA) with different preoperative data was investigated.

**Results:**

The mean CDVA change at 3 months postoperatively was −0.04 ± 0.19 and −0.07 ± 0.25 in the epi-on and epi-off groups, respectively (*p* = 0.809). In the epi-on group, this change was significantly correlated with the preoperative apical (*r* = −0.375, *p* = 0.045) and central corneal thickness (*r* = −0.402, *p* = 0.031). In the epi-off group, the CDVA change was significantly correlated with not only the preoperative apical (*r* = 0.402, *p* = 0.028) and central corneal thickness (*r* = 0.367, *p* = 0.046) but also with some topometric and aberrometric indices (*r* ≤ −0.374, *p* ≤ 0.042). Furthermore, the change in CDVA in the epi-on group could be predicted from age, preoperative refractive astigmatism J_45_ component, anterior corneal asphericity, and posterior corneal high order aberration root mean square (*p* = 0.002, *R*^2^ = 0.503). In the epi-off group, the CDVA change could be predicted from the preoperative minimum corneal thickness and magnitude of the vertical anterior corneal primary coma component (*p* = 0.001, *R*^2^ = 0.446).

**Conclusions:**

Clearly, different predictive factors of the visual change induced with the accelerated epi-on and epi-off CXL techniques are present, suggesting a different mechanism of action for stiffening the cornea and inducing changes in this structure.

## 1. Introduction

Keratoconus (KC) is a progressive disease in which the cornea becomes thinner, inducing irregular astigmatism and reduced quality of vision [1–3]. The exact mechanism of KC development is not well understood, but it is commonly accepted that genetic susceptibility and environmental factors are necessary [2]. In addition, secondary ectasia may be caused by a purely mechanical process in a predisposed cornea and may be unilateral [4]. The factors associated with KC include positive family history, atopic constitution, eye rubbing, sleep apnea, a place of living, blood group, and genetic syndromes, such as Down, chromosome translocation, and chromosome ring abnormality [1, 2].

The conservative treatment modalities, such as spectacles and gas permeable rigid contact lenses, become insufficient for visual rehabilitation in the advanced stages of KC, and 10–20% of patients need corneal transplantation [3]. Wollensak et al. [5] introduced the CXL treatment in 2003, which is being considered a standard, minimally invasive, and safe therapeutic option for progressive KC [5–18]. The principle goal of CXL is to stabilize the progression of KC by increasing the mechanical stability of the cornea [4–18]. Likewise, a successful CXL can prevent the progression of KC and can even cause the ectatic cornea to regress, but also a non-effect or even worsening of the ocular parameters can occur [2]. For this reason, CXL research in recent years has attempted to define the predictive factors for the outcomes achieved with this technique with the aim of helping the clinicians manage the patients' expectations and minimize the exposure to potential side effects. To date, multiple factors have been defined, including preoperative visual acuity, the eccentricity of the cone, pretreatment *K*_max_, age, and gender [2].

There is a significant variability in the few previous studies evaluating the predictive factors of the CXL outcome in KC [1, 2, 6]. This situation has increased the interest in recent years on this issue [6]. Peyman et al. [2] demonstrated that the lower pretreatment corneal asphericity and corneal keratoconus index (CKI) were associated to a higher *K*_max_ reduction after CXL [2]. Badawi et al. [6] observed that the preoperative best corrected visual acuity (BCVA) of more than 0.3 logMAR and *K*_max_ higher than 54 D were good predictors for post-CXL improvement in BCVA. Likewise, Wisse et al. [1] demonstrated that the eccentricity of the cone was the only predictor of keratometric changes during a 1-year follow-up after CXL [1].

For clinicians, the prediction of the effect of CXL treatment is a valuable tool to provide the patients with useful information about the postoperative course. The aim of the current study is to investigate the potential predictive factors of the epi-on and epi-off CXL outcomes in KC eyes, considering a great variety of clinical parameters and evaluating the differences in these predictive factors in the CXL techniques.

## 2. Methods

### 2.1. Study Population

The study was designed as a retrospective comparative study in which the medical records of 69 eyes of adult patients with progressive KC grades 1 to 4 (based on Amsler–Krumeich grading system) were revised. All of them had undergone the accelerated epithelial-on or accelerated epithelial-off CXL in the Torrecardenas University Hospital (Almeria, Spain) from May 2017 to January 2020. The classification of KC severity into four stages was performed in accordance with the Amsler–Krumeich grading criteria based on the mean corneal power, transparency, astigmatism, and the thinnest point of corneal thickness [19]. The research was carried out in accordance with the principles of the Declaration of Helsinki and was approved by the Clinical Research Ethics Committee of Torrecardenas Hospital (study code: FACCROSS-2021). Written informed consent was obtained from all patients before revising their medical records.

KC progression is defined as more than 1 D of steepening of maximum keratometry in the 6-month period before the surgery, an increase of more than 1.00 D in the manifest cylinder, or an increase of more than 0.50 D in the manifest refraction spherical equivalent in the previous twelve months, and a decrease of the corneal thickness by 30 *μ*m or more in the 6-month period before CXL surgery. The inclusion criteria were a biomicroscopic examination and a corneal topography consistent with KC, at least 18 years of age, the inferior-superior ratio on the topographic map of more than 1.5 D, the highest keratometric reading of 70 D or below, a minimum corneal thickness (MCT) of 400 *μ*m or higher, a clear cornea without scarring, and a minimum follow-up period of 12 months. Regarding the exclusion criteria, the following conditions were considered: corneal scarring, eyes with prior corneal surgery, other corneal diseases (e.g., herpetic keratitis and corneal opacities), serious medical conditions, malignancy, rheumatologic diseases, collagen vascular disease, hereditary connective tissue disorder (e.g., Marfan disease), severe dry eye, and pregnancy or lactation.

### 2.2. Clinical Examinations

The data collection of the study was divided into two parts. The first part included the collection of demographic data, such as age, gender, place of birth and residence, atopic constitution, family history (positive family history is defined as the existence of documented KC in first‐degree and second‐degree relatives), and eye rubbing history (evaluated using a 4-point Likert scale) [20]. The second part of the data collection consisted of extracting the information recorded in the ophthalmologic examinations performed during the follow-up in each patient, such as the grade of KC, preoperative uncorrected distance visual acuity (logMAR UDVA), and corrected distance visual acuity (logMAR CDVA) measured with a Snellen chart, slit-lamp biomicroscopy findings, tonometry, manifest refraction, dilated fundoscopic examination findings, keratometry, and topometric parameters. The topometric parameters included keratoconus index (KI), the index of surface variance (ISV), the index of height decentration (IHD), the index of vertical asymmetry (IVA), a minimum sagittal curvature (R_min_), an anterior average radius of curvature taken from the 3.0 mm optical zone centered on the thinnest point (ARC), and a posterior average radius of curvature taken from the 3.0 mm optical zone centered on the thinnest point (PRC). All this topographic information was obtained using Scheimpflug imaging-based topography device (Pentacam HR; Oculus GmbH, Wetzlar, Germany) that provides the anterior and posterior elevation data, as well as pachymetric, topometric, and keratometric data [8].

In all patients, the follow-up examinations were performed the next day, on the third day, and one week after the surgery to evaluate possible complications, epithelial scarring, and the absence of postoperative infection at 1, 3, and 12 months after the CXL surgery. In each examination, the manifest refraction, UDVA, CDVA, slit lamp biomicroscopic findings, corneal topography, and corneal thickness were recorded. Except for the visits up to one week after surgery, the assessment was with a slit lamp to assess the complications and epithelial scarring. For statistical analyses, the visual acuity data measured with a Snellen chart were converted to logMAR (logarithm of minimal angle of resolution) units.

### 2.3. Surgical Procedures

In all cases revised in this retrospective analysis, the CXL procedure had been performed under sterile conditions in an operating room under local anesthesia with 5% proparacaine HCl (Alcaine, Alcon). A speculum was placed on the eyelids after draping, and the steps followed according to the technique used were as follows:

#### 2.3.1. Epithelium-off Accelerated CXL

1. The placement of the patient in a supine position and the removal of corneal epithelium over the desired area.
2. The application of the solution Vibex Rapid (VibeX, Avedro Inc., Waltham, MA, USA; composition: 0.1% riboflavin, saline solution, and hydroxypropyl-methyl cellulose, HPMC) to completely wet and cover the exposed stroma. This combination diffuses twice as fast as dextran and minimizes corneal thinning, allowing a safer procedure.
3. The reapplication of this solution at least once every 2 minutes for a total of 10 minutes depending on the desired depth of crosslinking.
4. The rinsing of the eye with balanced saline solution (BSS) prior to irradiation.
5. The initiation of pulsed ultraviolet-A (UVA) treatment at 30 mW/cm^2^ for 8 minutes (1 second on, 1 second off) for a dose of 7.2 J/cm^2^.
6. The wetting of the cornea with BSS during UVA treatment as needed.

#### 2.3.2. Transepithelial (Epithelium-on) Accelerated CXL

1. The placement of the patient in a supine position without the removal of the corneal epithelium.
2. The application of Paracel Part One (VibeX, Avedro Inc., Waltham, MA, USA; composition: 0.25% riboflavin, benzalkonium chloride (BAC), ethylenediaminetetraacetic acid (EDTA), and HPMC) to completely cover the cornea and the repetition of this procedure every 90 seconds for 3.5 to 4 minutes. This solution loosens the epithelial junctions before the stroma is loaded with Paracel Part Two.
3. The rinsing of the cornea completely with Paracel Part Two (VibeX, Avedro Inc., Waltham, MA, USA; composition: 0.22% riboflavin, saline, isotonic).
4. The application of sufficient Paracel Part Two to completely cover the cornea and the repetition of this procedure every 90 seconds for 6 to 6.5 minutes (for a total riboflavin soaking time of 10 minutes).
5. The rinsing of the cornea completely with BSS.
6. The initiation of pulsed UVA at 45 mW/cm^2^ (higher irradiance needed because of UVA attenuation by the epithelium barrier) for 5 minutes and 20 seconds (1 second on, 1 second off) for a dose of 7.2 J/cm^2^.
7. The rinsing of the cornea completely with BSS. Patients were instructed not to rub the eye.

After the surgery, all patients were examined with a slit lamp on the 3^rd^ day and after one week to evaluate possible complications, epithelial healing, and the absence of postoperative infection. Topical moxifloxacin eye drops (Vigamox, Alcon, Fort Worth, Texas, USA) were prescribed to be applied 4 times daily for a period of one week, and artificial tears were to be applied 4 times daily for a period of one month. Fluorometholone 0.1% drop treatment (FML, Allergan, Dublin, Ireland) was initiated after epithelial healing in the epithelium-off patients with the application of drops 4 times daily for two weeks. Then, the dose frequency was tapering gradually. Soft contact lenses were fitted on the corneas postoperatively and removed after the corneal epithelium was fully cured, typically 3 to 5 days postoperatively.

### 2.4. Statistical Analysis

Statistical analysis was performed with SPSS program version 20 (SPSS, Chicago, IL). The descriptive statistical data were displayed as mean ± standard deviation (SD) for the continuous data and as a number with a percentage for the categorical data. The Kolmogorov–Smirnov test was used to check if the data distributions followed a normal distribution. The Chi-square test was utilized for the analysis of categorical variables. The student *t*-test for the unpaired data and the Mann–Whitney tests were used to analyze the differences between the epi-on and epi-off groups for data normally and not normally distributed, respectively. A *p*-value of less than 0.05 was considered statistically significant. The Spearman correlation coefficient was used to analyze the strength of the relationship among different variables within each group.

Besides these analyses, the multiple linear regression analysis (backward elimination method) was used to obtain a mathematical expression relating the change in CDVA after the CXL surgery with the preoperative variables for each technique, namely, epi-on and epi-off CXL. The analyses of residuals, homoscedasticity (the normality of unstandardized residuals), and influential points or outliers (Cook's distance) were performed to confirm the assumptions of the model obtained. Likewise, the Durbin–Watson test and the calculation of the variance inflation factor (VIF) were performed to assess the absence of correlation between the errors and multicollinearity.

## 3. Results

### 3.1. Demographic Characteristics

**Figure 1:**
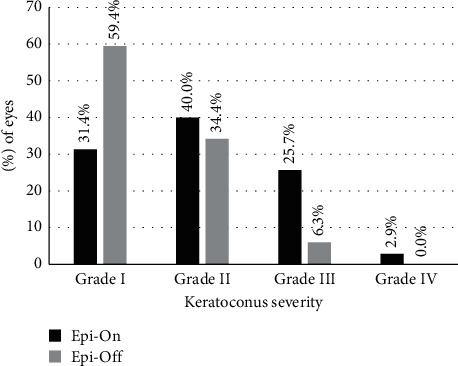
Distribution of keratoconus severity according to the Amsler–Krumeich grading system in the epi-on and epi-off groups.

This study included 69 patients (69 eyes) with KC who underwent CXL from May 2017 to January 2020. Of those, two patients did not have a complete follow-up and were excluded from the analysis (both undergoing epi-off CXL). Therefore, the data from 67 patients (67 eyes) with a complete follow-up of 12 months after CXL were analyzed. The sample included a total of 71.0% of males and 29.0% of females. The epi-on group consisted of a total of 35 eyes of 35 patients (50.7%), while the epi-off group comprised a total of 32 eyes of 32 patients (46.4%). The mean ages of the epi-on and epi-off groups were 26.5 ± 1.0 and 23.5 ± 1.0 years (*p* = 0.130) (Table 1). According to the Amsler–Krumeich grading system, a total of 30 KC eyes of grade I (43.5%), 26 eyes grade II (37.7%), 12 eyes grade III (17.4%), and 1 eye grade IV (1.4%) were included. No significant differences between the epi-on and epi-off groups were found in terms of keratoconus severity (*p* = 0.060) (Figure 1). A total of 23 patients (34.3%) were contact lens wearers during the follow-up period of this study. Specifically, 9 (25.7%) and 14 patients (43.8%) from the epi-on and epi-off groups were contact lens wearers (*p* = 0.173), respectively.

### 3.2. Visual and Refractive Changes

Table 2 shows the UDVA, CDVA, and manifest refraction data at the preoperative, 3-month postoperative, and 12-month postoperative visits. As shown, no significant differences between the epi-on and epi-off groups were found preoperatively in any visual and refractive parameters (*p* ≥ 0.166). Postoperatively, no significant differences were found between the epi-on and epi-off groups in the UDVA, CDVA, sphere, cylinder, spherical equivalent, and astigmatism power vectors (*p* ≥ 0.072).

### 3.3. Corneal Tomographic Changes

Table 3 shows the corneal tomographic data at the preoperative, 3-month postoperative, and 12-month postoperative visits. Significant differences between the epi-on and epi-off groups were found in different tomographic parameters: anterior flattest keratometry (K1) (*p* = 0.038), anterior steepest keratometry (K2) (*p* = 0.035), posterior K1 (*p* = 0.048), anterior maximum keratometry (K_max_) (*p* = 0.020), total deviation value (D index) (*p* = 0.002), central corneal thickness (CCT) (*p* = 0.004), minimal corneal thickness (MCT) (*p* < 0.001), and apex corneal thickness (ACT) (*p* = 0.001). Specifically, the eyes included in the epi-on group had corneas with more curvature and lower corneal thickness compared to the eyes from the epi-off group (Table 3). These significant differences found preoperatively in anterior K1, anterior K2, anterior K-max, D index, CCT, MCT, and ACT were also found at 3 months (*p* ≤ 0.028) and 12 months postoperatively (*p* ≥ 0.027). However, the difference between epi-on and epi-off groups did not reach statistical significance for posterior K1 at 3 (*p* = 0.137) and 12 months after the surgery (*p* = 0.129). Furthermore, at 12 months postoperatively, a significantly lower corneal volume was found in the epi-on group compared to the epi-off group (*p* = 0.044).

### 3.4. Changes in Topometric Indices

Table 4 shows the topometric indices measured at the preoperative, 3-month postoperative, and 12-month postoperative visits. Significant differences between the epi-on and epi-off groups were found in all preoperative parameters: IHD (*p* = 0.005), ISV (*p* = 0.012), IVA (*p* = 0.029), KI (*p* = 0.037), ARC (*p* = 0.018), and PRC (*p* = 0.010). At 3 months postoperatively, significant differences between the groups were found in IHD (*p* = 0.031), ISV (*p* = 0.015), KI (*p* = 0.035), and R_min_ (*p* = 0.020). Likewise, statistically significant differences between the epi-on and epi-off groups were at 12 months after the surgery in ISV (*p* = 0.035), R_min_ (*p* = 0.020), ARC (*p* = 0.012), and PRC (*p* = 0.020).

### 3.5. Corneal Aberrometric Changes

Table 5 shows the corneal aberrometric data measured at the preoperative, 3-month postoperative, and 12-month postoperative visits. Statistically significant differences were found between the epi-on and epi-off groups in the preoperative aberrometric variables: anterior (*p* = 0.013) and posterior (*p* = 0.010) total RMS, anterior HOA RMS (*p* = 0.042), and anterior (*p* = 0.014) and posterior (*p* = 0.013) LOA RMS. At 3 months after surgery, significant differences between the epi-on and epi-off groups were found in the same variables as preoperatively (*p* ≤ 0.044) and in posterior HOA RMS (*p* = 0.005). At 12 months postoperatively, these significant differences were maintained only in the anterior total RMS (*p* = 0.045), anterior (*p* = 0.048) and posterior (*p* = 0.048) HOA RMS, and anterior LOA RMS (*p* = 0.048).

### 3.6. Correlation of Visual Changes with Preoperative Variables

The mean change at 3 months after the surgery in logMAR CDVA was −0.04 ± 0.19 and −0.07 ± 0.25 in the epi-on and epi-off groups, respectively (*p* = 0.809). At 12 months postoperatively, the mean change in logMAR CDVA was −0.07 ± 0.19 and −0.16 ± 0.23 in the epi-on and epi-off groups, respectively (*p* = 0.087). In the epi-on group, the mean 3-month change in CDVA was significantly correlated with the preoperative apical corneal thickness (*r* = −0.375, *p* = 0.045), central corneal thickness (*r* = −0.402, *p* = 0.031), and anterior corneal asphericity (*r* = −0.363, *p* = 0.050). In the epi-off group, the mean 3-month change in CDVA was significantly correlated with the preoperative apical corneal thickness (*r* = 0.402, *p* = 0.028), central corneal thickness (*r* = 0.367, *p* = 0.046), ISV (*r* = −0.405, *p* = 0.027), IVA (*r* = −0.394, *p* = 0.031), anterior total RMS (*r* = −0.374, *p* = 0.042), and anterior LOA RMS (*r* = −0.374, *p* = 0.042).

In the epi-on group, no correlation of the visual change at 3 (*r* = −0.084, *p* = 0.664) and 12 months (*r* = −0.043, *p* = 0.835) after surgery with age was found. Likewise, no significant differences in the visual change at 3 (−0.03 ± 0.15 vs. −0.06 ± 0.26, *p* = 0.649) and 6 months (−0.06 ± 0.16 vs. −0.09 ± 0.25, *p* = 0.874) were found between the patients not wearing contact lenses and those wearing them. Similarly, in the epi-off group, no correlations of age with 3-month (*r* = −0.146, *p* = 0.426) and 12-month (*r* = -0.059, *p* = 0.749) visual changes were found. Furthermore, in the same group, no significant differences in the 3-month (−0.04 ± 0.18 vs. −0.16 ± 0.34, *p* = 0.343) and 12-month (−0.10 ± 0.14 vs. −0.28 ± 0.30, *p* = 0.116) visual changes were found between the users not using the contact lens and those using them.

### 3.7. Multiple Linear Regression Analysis

In the epi-on group, a statistically significant linear relationship between the 3-month changes in CDVA (ΔCDVA) was obtained according to the following expression (*p* = 0.002, *R*^2^ = 0.503, adjusted *R*^2^: 0 : 420, Durbin–Watson: 2.207):

(1) $$\begin{array}{c} \Delta \text{CDVA}=0.063-0.006\times \text{Age}-0.075\times {\text{J}}_{45}-0.203\times \text{Q}-\text{val}-0.140\times {\text{HOA}}_{\text{post}}\text{RMS,} \end{array}$$

where J_45_ is one of the refractive astigmatism power vectors, Q-val is the anterior corneal asphericity, and HOA_post_ RMS is the posterior higher order aberration root mean square.

The normality of the unstandardized residuals distribution (*p* = 0.200) and the absence of influential points or outliers (mean Cook's distance = 0.045 ± 0.076) confirmed the homoscedasticity of this model. Likewise, no multicollinearity was detected in the model (variance inflation factor between 1.068 and 1.283).

In the epi-off group, a statistically significant linear relationship between the 3-month changes in CDVA (ΔCDVA) was obtained according to the following expression (*p* = 0.001, *R*^2^ = 0.446, adjusted *R*^2^: 0 : 398, Durbin–Watson: 2.200):

(2) $$\begin{array}{c} \Delta \text{CDVA}=-1.551+0.003\times \text{MCT}+0.171\times Z_{3}^{-1}, \end{array}$$

where MCT is the minimal corneal thickness and *Z*_3_^−1^ is the Zernike term corresponding to the anterior vertical primary coma.

The normality of the unstandardized residuals distribution (*p* = 0.200) and the absence of influential points or outliers (mean Cook's distance = 0.044 ± 0.080) confirmed the homoscedasticity of this model. Likewise, no multicollinearity was detected in the model (variance inflation factors 1.073).

## 4. Discussion

Corneal CXL, as a photooxidative procedure, enhances the mechanical stability of the cornea because of the synthesis of the well-structured collagen and new lamellar interconnections in the cornea [5, 21–23]. The promising results of CXL in the management of either KC or corneal ectasia have encouraged the researchers to consider it one of the substantial initial treatment procedures for these conditions [24, 25]. Few studies with variable results have been conducted to date to define the potential predictive factors for the clinical outcome obtained with this surgical procedure in terms of visual or corneal tomographic changes or even in terms of the progression of KC [26–29]. There are clinical studies proving additional insights into the factors associated with the CXL outcomes in KC patients [1–3, 6]. However, the preoperative predictors of the visual changes after CXL were not entirely illustrated, and there is still a necessity for further research on this issue. In this study, the predictive factors for the visual change induced after the two different techniques of CXL, accelerated epi-on and epi-off, have been investigated.

In the present study, the visual and refractive results obtained with the two CXL techniques did not differ significantly at the preoperative and postoperative visits, suggesting that both techniques were comparable in terms of visual and refractive outcomes and potentially in the ability of halting the progression of keratoconus during a 1-year follow-up. Likewise, the visual acuity outcomes of our study are consistent and comparable to other CXL reports for the treatment of progressive corneal ectasia [9, 30–38]. Shalchi et al. [39] revised the clinical outcomes of 45 articles that evaluated the CXL epithelium-off technique and six articles that evaluated the transepithelial CXL technique. These authors could not perform a metaanalysis with all the data because there was only one randomized, controlled clinical trial comparing the two CXL techniques. This particular trial evaluated only the corneal morphological changes on the confocal microscopy and optical coherence tomography and did not report the corneal topography or visual outcomes [39]. Using the published data in the review article of Shalchi et al. [39], a mean change in the CDVA of −0.08 ± 0.07 logMAR (range, −0.23 to +0.06) was calculated for the 45 articles evaluating the epi-off CXL technique. For the six articles evaluating the transepithelial technique, a mean change in the CDVA of −0.08 ± 0.04 logMAR (range, −0.12 to −0.04) was calculated. It is important to note that in our study, the data of epi-on accelerated CXL have been analyzed in comparison to the review of Shalchi et al. [39] that analyzes the articles reporting the outcomes of epi-on standard CXL surgery. In the current sample, the mean changes in the CDVA of −0.06 ± 0.19 (range, −0.14 to 0.01) and −0.16 ± 0.23 (range, −0.24 to −0.07) were found at 1 year after the accelerated epi-on and epi-off CXL, respectively.

In our study, we observed in the two groups that the anterior K2, K-max, CCT, MCT, and ACT showed improvement at one year of follow-up, although the magnitude of the change achieved was relatively small (Table 3). Rossi et al. showed that the treatments epi-on and epi-off stopped the progression of keratoconus in all eyes from their comparative study over a 12-month period [40]. Magli et al. compared epi-on vs epi-off and observed similar results in a study with pediatric patients under 18 years of age [41]. In contrast, some studies have found that the patients treated with epi-on CXL had a greater KC progression than those treated with epi-off CXL [42]. Kocak et al. [43] reported a stabilization of the corneal ectasia in 89% of the eyes treated with epi-off CXL, whereas only in 35% of the eyes treated with epi-on CXL. Cerman et al. [44] found that 97% of the epi-off eyes achieved stabilization, whereas 80% of the epi-on eyes achieved stabilization in a comparative study, although these authors associated greater ectasia progression in the epi-on eyes to the presence of more cases of advanced and progressive KC disease in such group. Despite the differences in the results, the four studies found a faster visual recovery and a reduction of pain and infections related to the epithelial defects in the epithelium-on groups. The mean preoperative and postoperative *K*_max_ (D) values in the epi-on group in different comparative studies were 52.41 ± 5.39 and 50.5 ± 5.37 D, [40] 49.27 ± 4.1 and 48.13 ± 5.4 D, [41] 48.75 ± 6.82 and 50.57 ± 6.82 D, [43] 60.12 ± 6.17 and 60.0 ± 6.31 D, [44] and 54.7 ± 4.0 and 53.7 ± 3.7 D, [45] respectively. Caruso et al. [46] observed a reduction in the mean *K*_max_ value of −1.10 ± 1.22 D at the end of the follow-up after epi-on CXL. In our study, the mean preoperative and postoperative *K*_max_ values in the epi-on group were 60.31 ± 6.47 and 59.88 ± 7.19 D, respectively. With respect to the mean values of CCT (microns) in the epi-on group in different comparative studies, the preoperative and postoperative values of 451 ± 39.51 and 448.4 ± 37.32 *μ*m, [40] 490.2 ± 22.3 and 488.0 ± 19.3 *μ*m, [41] 470 ± 38 and 446 ± 59 *μ*m, [43] and 484 ± 37 and 491 ± 27 *μ*m [45] have been reported, respectively. Cerman et al. [44] analyzed MCT changes in a study with 18 months of follow-up, finding the mean values of 425.3 ± 22.4 and 419.4 ± 24.3 *μ*m before and at the end of the follow-up, respectively. Caruso et al. [46] observed a mean increase in the MCT of 6.6 ± 24.0 *μ*m at the end of the follow-up. In our study, the mean CCT and MCT values in the epi-on group were 455.64 ± 36.47 and 458.78 ± 35.88 *μ*m preoperatively and 428.70 ± 35.36 and 433.07 ± 35.02 *μ*m at the end of the follow-up.

Besides these investigations, other CXL protocols have been evaluated that are based on the epi-on concept, such as the CXLO protocol that uses a new sodium iodide riboflavin formulation that theoretically allows a greater level of penetration [47–51]. These investigations have shown that the use of the CXLO protocol can halt the progression of ectasia and result in better visual acuity without the risk associated to epi-off CXL [47–51]. Three of these studies show the results of this type of CXL applied one day after conductive keratoplasty (CK) [47–49]. Sinjab et al. [49] observed at the end of the follow-up a mean reduction of *K*_max_ with the combination of CK and CXLO of 3.8 D with the mean baseline *K*_max_ value of 65.1 ± 11 D. Rechichi et al. [52] evaluated the changes in the refraction and corneal aberrations in keratoconus after the selective transepithelial topography-guided photorefractive keratectomy combined with accelerated corneal crosslinking (STARE-X) and demonstrated effective results to stop the progression of keratoconus. Specifically, there was an improvement of visual acuity and corneal aberration and a statistically significant reduction in *K*_max_ at 2 years after surgery.

In the current sample, the mean changes in *K*_max_ were −0.45 ± 1.96 D and −1.05 ± 1.56 D at 1 year after surgery in the epi-on and epi-off CXL groups, respectively. Likewise, the mean changes in MCT were 3.75 ± 1.56 *μ*m and 2.41 ± 1.69 *μ*m at 1 year after the surgery in the epi-on and epi-off groups, respectively. These results should be analyzed considering that the percentage of moderate and advanced keratoconus (grade III and IV) was 28.6% and 6.3% in the epi-on and epi-off groups, respectively. Thus, in our study, the patients included in the accelerated epi-on group tended to have more severe keratoconus than in the accelerated epi-off group, presenting a higher baseline K-max and K2 and lower ACT, CCT, and MCT values. It can be explained by the fact that in our hospital, the epi-on techniques are preferred on the thinner corneas that are normally present in more severe keratoconus for protecting the corneal endothelium from excessive riboflavin penetration and UV radiation. Considering this situation (worse baseline conditions in the epi-on group), better results were expected at the end of the follow-up in the epi-off group. However, the differences in the visual results did not reach the statistical significance, while the discrepancies between the groups in terms of corneal tomographic and topographic data were maintained. Sloot et al. [53] demonstrated that the amount of flattening by CXL was directly proportional to the steepness of the cornea. However, in our series, the results of the epi-on CXL were not inferior to those obtained with the epi-off CXL technique at 1 year after the surgical procedure was done despite the significantly worse baseline conditions of the patients in the epi-on group.

With respect to the corneal volume, statistically significant (*p* = 0.044) differences between the epi-on and epi-off groups were only found at 12 months after the surgery (epi-on 56.01 ± 3.75 mm^3^ vs. epi-off 58.16 ± 4.23 mm^3^). However, the corneal volume also tended to be lower in the epi-on group preoperatively and at 3 months after the surgery, which is consistent with the more reduced measures of corneal thickness (ACT, CCT, and MCT) obtained in this group at all visits. The lower corneal volume in the epi-on group compared to the epi-off group is because of the inclusion of more cases of moderate-to-advanced keratoconus in that group as a greater reduction in the corneal volume is present in the eyes with more advanced keratoconus [38]. In the current study, an analysis of the corneal biomechanical variables was not performed. However, in another study, the parameters of corneal hysteresis (CH) and corneal resistance factor (CRF) were found to be correlated with morphogeometric and volumetric parameters in the corneas with keratoconus. This correlation was, however, highly influenced by the thickness of the cornea [54].

In the epi-on group, more patients with moderate-to-advanced keratoconus were included, and the presence of high-order aberrations was higher as they had a more altered and irregular cornea. Indeed, significant differences between the groups were found preoperatively and postoperatively in other indices characterizing the level of corneal irregularity, such as IHD, ISV, or KI. It should be considered that Kanellopoulos and Asimellis [55] concluded that ISV and IHD may be the most sensitive and specific criteria in the diagnosis and progression of keratoconus. The anterior corneal HOAs and LOAs showed a trend toward improvement in both groups, as in the other previous studies [8, 56]. However, the difference between the groups was maintained during the follow-up in most of the aberrometric parameters. It should be considered that this aberrometric change was associated with a visual improvement in both groups, as in the previous series evaluating the outcomes of different CXL techniques [8, 24, 25, 57]. Ghanem et al. [58] concluded that the improvement in the high-order aberrations in keratoconus patients was attributed to the flattening of the corneal apex caused by the CXL effect. In contrast to what happens in the anterior corneal surface after CXL, a trend toward the worsening of the posterior high-order aberrations RMS was found after both techniques, epi-on and epi-off, maintaining the differences between the groups during the follow-up. This could be explained by the significant changes that occur in the mechanical resistance of the anterior cornea as a consequence of the CXL procedure and this probably produces changes in the geometry of the posterior corneal surface. A similar finding was reported in a previous study evaluating the posterior aberrometric impact of the accelerated epi-on CXL surgery [59]. In the same study, significant steepening and change to the significant prolateness was observed in these patients undergoing epi-on CXL [59].

Regarding the safety of CXL, we observed in the epi-off group one case of sterile infiltrates that were successfully solved with topical corticosteroids, without leaving sequelae. In the epi-off group, only one case of small epithelial defects that completely closed on the 3^rd^ postoperative day was reported. No cases of infectious keratitis or stromal scars were reported in any patient from both groups. Koller et al. [26] and Ghanem et al. [60] reported sterile infiltrate rates after the epi-off CXL in 7.6% and 0.97% of the eyes in their series, respectively. Stulting et al. [50] reported a rate of 5% for small epithelial defects the day after treatment, however, almost all were closed the next day. Furthermore, in our study, stromal haze was present in 8.8% of the patients in the epi-off group (3 patients), which was minimized at around 6 months in all patients. The incidence of postoperative corneal haze after the epi-off CXL varies significantly from the studies, with a variation from 10% [8, 61] to values over 20% [62].

In the epi-on group, the mean 3-month change in CDVA was significantly correlated with the preoperative ACT, CCT, and anterior corneal asphericity. Specifically, these correlations revealed that more visual improvement was expected in those eyes with less prolate and thicker corneas. In contrast, in the epi-off group, the mean 3-month change in CDVA was found to be significantly correlated with the preoperative ACT, CCT, ISV, IVA, anterior total RMS, and anterior LOA RMS, with more improvement expected in the thinner corneas and with more anterior aberrations and higher values of ISV and IVA at the baseline. It confirms that the predictive factors for the visual change induced with the two techniques of CXL evaluated were clearly different, and consequently, it confirms that the differential mechanisms of action were present with both techniques. The previous studies investigated some potential predictive factors for the effect of epi-off CXL but not for the effect of epi-on CXL. Some of them are consistent with those found in the epi-off group in the current series. Wisse et al. [1] confirmed that the cone eccentricity was the sole predictor of keratometry outcomes at 1 year after epi-off CXL, demonstrating also that pretreatment CDVA could be used to reliably predict CDVA 1 year after treatment. Toprak et al. [3] observed that a preoperative CDVA <20/40 was significantly related to postoperative visual improvement after epi-off CXL. Likewise, these authors confirmed that the corneas with the thinnest corneal pachymetry below 450 *μ*m experienced significantly more flattening in the maximum K. In the study conducted by Greenstein and Hersh, the only independent predictor of the change in postoperative CDVA after CXL was the preoperative CDVA [28]. Badawi et al. [5] demonstrated that a higher K-max, worse CDVA, and relatively thinner corneas were associated with a greater improvement after epi-off CXL, whereas Peyman et al. [2] observed that a lower pretreatment logMAR CDVA, thinner pretreatment CCT, and a higher pretreatment KI was associated with higher postoperative logMAR CDVA reduction. In addition, Koller et al. [27] observed that a higher baseline K‐max was associated with a greater degree of flattening.

Besides the analysis of the correlation of the visual change in each group with the preoperative data, a multiple linear regression analysis was performed to obtain a linear expression allowing the clinician to obtain an estimation of the visual change expected after the epi-on and epi-off CXL techniques. Likewise, the validity of the two predictive models obtained was investigated. The analysis revealed that the visual change induced by the epi-on CXL techniques could be predicted from the following preoperative variables: age, the oblique component of refractive astigmatism (J_45_), anterior corneal asphericity, and the RMS value of the posterior corneal high-order aberrations. In contrast, in the epi-off group, other different variables were involved in the predicting equation: preoperative MCT and Zernike term corresponding to the vertical coma of the anterior corneal surface. It confirms, as happened in the correlation analysis, the presence of differential factors for the prediction of the visual change after epi-on and epi-off CXL, suggesting the presence of clearly different mechanisms of action. Specifically, age was only found to be a critical factor of the prediction equation of the visual change induced in the epi-on group despite an extremely weak correlation found between age and visual change in both epi-on and epi-off groups. It may be related to the different mechanism of action and level of penetration with epi-on CXL, leading to differences in the response of the treatment in those eyes with weaker corneas or with more predisposition to progression. It has been previously reported that the factors possibly contributing to a potentially lesser response of CXL with the transepithelial approach include alterations of the epithelium and basement membrane, epithelial absorption of ultraviolet A radiation, and other patient variables, such as refraction, *K*_max_, and pachymetry [14]. The riboflavin solution used in the transepithelial study group was formulated with benzalkonium chloride 0.01%, which has been reported to enhance the ocular surface penetration by increasing the epithelial permeability and bioavailability of topical medication to the corneal stroma [63]. Rush and Rush [9] concluded that the ocular surface penetration of riboflavin into the corneal stroma in a transepithelial CXL study group was inferior to that in the epithelium-off control group despite the application of a more concentrated riboflavin formulation and the frequent instillation of benzalkonium chloride. However, a different level of penetration does not necessarily mean a lower capability of controlling keratoconus progression or less visual impact. It can only be related to a different mode of modifying the mechanical properties of the cornea and generating clinical changes. Indeed, no correlation has been found between the demarcation line depth (level of penetration of riboflavin) and visual or topographic outcomes after the epi-on and epi-off CXL techniques [64]. More research is needed in the future to understand better the exact mechanism of action of each CXL technique to strengthen the corneal structure.

The multiple linear regression analysis revealed that more significant visual improvement was present at 3 months after epi-on CXL in younger people with higher preoperative levels of posterior HOA RMS, less anterior corneal prolateness, and more positive J_45_ refractive component. In contrast, more significant visual improvement was expected at 3 months after epi-off CXL in those eyes with thinner corneas and significant levels of primary coma on the anterior corneal surface. Therefore, as suggested in the previous series, [1–3, 5, 27, 28] more visual improvement was expected in those eyes with moderate-to-severe keratoconus when using epi-off CXL but not when using epi-on CXL. With the epi-on CXL technique, the visual change induced with surgery does not seem to be related to the level of severity of keratoconus. This aspect should be considered when selecting the type of CXL to apply in each case, being more recommendable in terms of potential visual improvement of the epi-off technique in moderate to advanced keratoconus. In any case, more studies in other keratoconus populations should be conducted to validate and refine these two models of prediction of the visual change after CXL. Finally, it should be mentioned that the use of contact lens was not found to be a critical or confounding factor in these predicting equations, confirming its minimal influence on the outcomes obtained. Furthermore, it should be considered that only a minor portion of the patients were contact lens wearers in the current sample.

Our study had several limitations that must be mentioned and acknowledged. Firstly, more cases of moderate-to-advanced keratoconus were included in the epi-on group, and more future comparative studies must be performed including more comparable samples in terms of keratoconus severity. Secondly, although this study provides useful information about proper patient selection according to the CXL technique, the predictors proposed should be used with care as they cannot be generalizable to all patients, especially to those falling outside the range of the study population. Therefore, this study should be validated by future studies for a proper implementation in clinical practices. Thirdly, the retrospective design might be considered a limitation of the study. It should be considered that the information about atopy and familiarity were not available in most of the clinical histories revised, and therefore, its role as a potential confounding factor was not investigated.

In conclusion, the changes in logMAR CDVA at 3 months after epi-on CXL were significantly associated with the preoperative magnitude of the posterior HOA RMS, age, level of anterior corneal prolateness, and the J_45_ refractive component. In contrast, the visual changes after epi-off CXL were significantly associated with the corneal thickness and the level of primary coma present on the anterior corneal surface. These significant differences in the predictive factors of the visual change associated with each CXL technique suggests that the mechanism of action for modifying the mechanical strength of the cornea may differ significantly. There is no clear benefit for a 1-year follow-up of one CXL technique over the other, however, more visual improvement might be expected in more severe keratoconus cases. These factors can provide new insights into the mechanism of action of each CXL technique and provide new insights into the prediction of the CXL effect for better patient selection. However, more studies are needed to validate and refine these predictive models.

## Figures and Tables

**Table 1:** Demographic data of the patients enrolled in study.

*CXL type*	Epi-on 35 (50.7%)
	Epi-off 34 (49.3%)

*Sex*	49 males (71.0%)
	20 females (29.0%)

*Age (years)*	24.9 ± 1.0
	Epi-on 26.5 ± 1.0
	Epi-off 23.5 ± 1.0

*Laterality*	36 right eyes (52.2%)
	33 left eyes (47.8%)

*Contact lenses*	No 46 (66.7%)
	Yes 23 (33.3%)

**Table 2:** Differences between the epi-on and epi-off groups in the visual and refractive parameters in the different visits of the follow-up.

Mean (SD) Median (Range)	Preoperative		*p*-value	Postoperative 3 months		*p*-value	Postoperative 12 months		*p*-value
	Epi-on	Epi-off		Epi-on	Epi-off		Epi-on	Epi-off	
UDVA (logMAR)	0.75 (0.44)	0.75 (0.38)	0.741	0.67 (0.42)	0.73 (0.40)	0.620	0.65 (0.36)	0.66 (0.42)	0.993
	0.7 (1.90)	0.82 (1.25)		0.82 (1.25)	0.70 (1.25)		0.70 (1.25)	0.61 (1.30)	
Sphere (D)	−0.75 (1.70)	−1.58 (3.14)	0.166	−0.67 (2.11)	−1.53 (3.36)	0.396	−0.73 (1.78)	−1.57 (2.94)	0.366
	0.00 (8.00)	−0.75 (16.00)		0.00 (0.75)	−0.50 (17.00)		−0.13 (9.00)	−0.68 (14.00)	
Cylinder (D)	−3.35 (1.75)	−3.01 (1.50)	0.584	−2.61 (2.08)	−2.35 (1.75)	0.983	−2.74 (1.96)	−2.39 (1.59)	0.542
	−3.00 (8.00)	−3.00 (5.50)		−2.00 (8.00)	−2.50 (6.00)		−2.50 (9.25)	−2.37 (7.00)	
SE (D)	−2.42 (1.95)	−3.09 (3.13)	0.384	−1.98 (2.41)	−2.71 (3.57)	0.335	−2.11 (2.11)	−2.77 (3.22)	0.170
	−2.00 (9.50)	−2.37 (15.63)		−1.25 (10.75)	−1.63 (18.50)		−1.62 (11.13)	−1.70 (17.00)	
J_0_ (D)	−0.02 (1.34)	−0.21 (1.26)	0.485	−0.39 (1.24)	−0.31 (1.08)	0.917	−0.25 (1.30)	−0.21 (0.95)	0.998
	0.00 (5.45)	−0.23 (4.52)		−0.62 (6.35)	−0.21 (4.95)		−0.50 (6.35)	−0.21 (3.87)	
J_45_ (D)	0.17 (1.35)	0.17 (1.11)	0.772	0.05 (1.06)	0.10 (0.95)	0.706	0.19 (1.12)	0.33 (1.02)	0.505
	0.00 (5.93)	−0.16 (3.80)		0.00 (4.50)	0.00 (3.96)		0.04 (4.74)	0.16 (4.97)	
CDVA (logMAR)	0.36 (0.23)	0.33 (0.28)	0.322	0.31 (0.23)	0.27 (0.15)	0.630	0.28 (0.23)	0.16 (0.14)	0.072
	0.30 (1.00)	0.30 (1.30)		0.30 (0.70)	0.30 (0.70)		0.26 (0.82)	0.15 (0.52)	

UDVA, uncorrected distance visual acuity; logMAR, logarithm of minimal angle of resolution; SD, standard deviation; SE, spherical equivalent; CDVA, corrected distance visual acuity; J_0_ and J_45_, astigmatism power vectors; D, diopters.

**Table 3:** Differences between the epi-on and epi-off groups in the corneal tomographic parameters in the different visits of the follow-up.

Mean (SD)	Preoperative		*p*-value	Postoperative 3 months		*p*- value	Postoperative 12 months		*p*- value
Median (Range)	Epi-on	Epi-off		Epi-on	Epi-off		Epi-on	Epi-off	
Anterior K1 (D)	46.90 (3.93)	45.02 (3.22)	0.038	47.20 (3.81)	45.05 (3.34)	0.021	47.07 (4.20)	44.75 (3.52)	0.027
	47.50 (14.70)	44.40 (12.80)		47.70 (13.50)	44.60 (12.10)		47.55 (16.60)	44.20 (12.40)	
Anterior K2 (D)	51.93 (4.87)	49.52 (4.18)	0.035	51.71 (4.68)	49.15 (4.07)	0.024	51.88 (5.36)	48.89 (4.34)	0.021
	52.25 (19.80)	49.25 (18.30)		52.70 (19.30)	49.25 (17.30)		52.01 (20.10)	48.60 (17.80)	
Posterior K1 (D)	−6.94 (0.83)	−6.55 (0.72)	0.048	−6.90 (0.77)	−6.61 (0.73)	0.137	−6.91 (0.83)	−6.60 (0.71)	0.129
	−6.85 (3.30)	−6.50 (2.70)		−7.00 (2.60)	−6.55 (2.90)		−7.00 (3.60)	−6.50 (2.90)	
Posterior K2 (D)	−7.92 (1.00)	−7.53 (0.84)	0.092	−7.95 (0.98)	−7.50 (0.84)	0.054	−7.86 (1.02)	−6.75 (3.14)	0.085
	−7.90 (3.80)	−7.55 (3.60)		−8.00 (3.90)	−7.45 (3.60)		−7.95 (3.90)	−7.50 (17.20)	
K-max (D)	60.31 (6.47)	56.54 (6.35)	0.020	59.90 (6.24)	56.15 (6.27)	0.028	59.88 (7.19)	55.49 (6.62)	0.018
	59.50 (26.50)	55.60 (23.40)		60.35 (25.70)	55.75 (23.30)		59.85 (28.50)	54.30 (25.30)	
D index	11.26 (4.09)	8.31 (3.36)	0.002	11.54 (4.09)	8.38 (3.18)	0.001	10.96 (4.55)	8.23 (3.32)	0.011
	11.49 (16.53)	7.82 (13.52)		11.81 (15.67)	8.53 (14.35)		11.44 (16.85)	8.39 (13.97)	
CCT (*μ*m)	455.64 (36.47)	484.34 (42.16)	0.004	460.58 (38.34)	485.09 (41.67)	0.015	458.78 (35.88)	488.54 (40.02)	0.004
	454.38 (135.00)	476.0 (157.00)		447.00 (142.00)	476.00 (155.00)		460.00 (140.00)	477.00 (149.00)	
ACT (*μ*m)	442.47 (38.91)	477.03 (41.89)	0.001	446.00 (39.79)	478.59 (42.17)	0.002	447.00 (36.59)	480.64 (42.05)	0.003
	436.00 (135.00)	468.00 (158.00)		435.00 (129.00)	469.00 (167.00)		443.50 (132.00)	469.00 (145.00)	
MCT (*μ*m)	428.70 (35.36)	467.09 (41.08)	<0.001	430.61(37.08)	468.75(42.58)	0.001	433.07 (35.02)	470.19 (42.46)	0.001
	419.50 (141.0)	461.0 (157.0)		425.0(158)	461.0(170)		428.50 (126.0)	463.0 (145.0)	
Corneal volume (mm^3^)	55.62 (3.47)	57.21 (4.11)	0.094	56.14 (3.63)	57.70 (4.09)	0.114	56.01 (3.75)	58.16 (4.23)	0.044
	55.65 (16.40)	57.90 (16.60)		56.40 (17.40)	59.00 (16.00)		56.30 (16.60)	59.10 (16.70)	
Q-val	−0.94 (0.53)	−0.78 (0.43)	0.187	−0.94 (0.55)	−0.77 (0.46)	0.192	−0.91 (0.51)	−0.69 (0.41)	0.064
	−0.85 (2.52)	−0.80 (1.62)		−0.99 (2.26)	−0.73 (2.02)		−0.97 (1.98)	−0.68 (1.47)	

K1, flattest keratometry; K2, steepest keratometry; K-max, maximum keratometry; D, total deviation value; CCT, central corneal thickness; ACT, apex corneal thickness; Q-val, asphericity of the anterior corneal surface; MCT: minimal corneal thickness.

**Table 4:** Differences between the epi-on and epi-off groups in the corneal topometric parameters in the different visits of the follow-up.

Mean (SD)	Preoperative		*p*-value	Postoperative 3 months		*p*-value	Postoperative 12 months		*p*-value
Median (Range)	Epi-on	Epi-off		Epi-on	Epi-off		Epi-on	Epi-off	
IHD	0.20 (0.14)	0.13 (0.07)	0.005	0.19 (0.12)	0.13 (0.07)	0.031	0.21 (0.16)	0.17 (0.15)	0.191
	0.18 (0.88)	0.12 (0.35)		0.18 (0.67)	0.13 (0.32)		0.18 (0.67)	0.13 (0.71)	
ISV	114.11 (34.63)	89.78 (41.90)	0.012	113.55 (36.10)	91.81 (36.63)	0.015	110.25 (40.96)	88.61 (39.80)	0.035
	110.00 (149.00)	84.50 (193.00)		110.00 (144.00)	91.00 (180.00)		106.50 (147.00)	86.00 (191.00)	
IVA	1.23 (0.47)	0.94 (0.59)	0.029	1.21 (0.49)	0.98 (0.53)	0.087	1.17 (0.52)	0.96 (0.56)	0.161
	1.23 (1.72)	0.87 (2.76)		1.14 (1.72)	0.98 (2.52)		1.15 (1.64)	0.95 (2.65)	
KI	1.32 (0.14)	1.24 (0.15)	0.037	1.31 (0.14)	1.24 (0.14)	0.035	1.31 (0.16)	1.23 (0.14)	0.058
	1.33 (0.64)	1.22 (0.75)		1.33 (0.65)	1.23 (0.69)		1.29 (0.61)	1.24 (0.74)	
R_min_ (mm)	5.66 (0.62)	6.03 (0.66)	0.021	5.67 (0.61)	6.05 (0.63)	0.020	5.71 (0.69)	6.15 (0.70)	0.020
	5.67 (2.70)	6.07 (2.46)		5.58 (2.67)	6.06 (2.34)		5.64 (2.85)	6.22 (2.67)	
ARC (mm)	6.32 (0.63)	6.68 (0.55)	0.018	6.25 (0.59)	6.74 (0.53)	0.108	6.36 (0.65)	6.80 (0.61)	0.012
	6.09 (2.40)	6.60 (2.04)		6.08 (2.42)	6.68 (2.04)		6.09 (2.37)	6.71 (2.34)	
PRC (mm)	4.65 (0.58)	5.02 (0.56)	0.010	4.56 (0.52)	5.03 (0.50)	0.095	4.69 (0.63)	5.05 (0.52)	0.020
	4.52 (2.44)	4.98 (2.17)		4.53 (2.19)	4.98 (1.91)		4.59 (2.52)	5.01 (2.09)	

IHD, index of height decentration; ISV, index of surface variance; IVA, index of vertical asymmetry; KI, keratoconus index; R_min_, minimum radii of curvature; ARC, anterior average radius of curvature taken from the 3.0 mm optical zone centered on the thinnest point; PRC, posterior average radius of curvature taken from the 3.0 mm optical zone centered on the thinnest point.

**Table 5:** Differences between epi-on and epi-off groups in corneal topometric parameters in the different visits of the follow-up.

Mean (SD)	Preoperative		*p*- value	Postoperative 3 months		*p*- value	Postoperative 12 months		*p*- value
Median (Range)	Epi-on	Epi-off		Epi-on	Epi-off		Epi-on	Epi-off	
Anterior total RMS (*μ*m)	15.84 (5.35)	12.93 (6.10)	0.013	15.63 (5.38)	12.30 (5.73)	0.018	15.00 (6.19)	11.71 (6.14)	0.045
	15.14 (22.97)	12.33 (28.58)		15.01 (22.33)	12.09 (26.45)		13.71 (23.21)	11.44 (28.01)	
Anterior HOA RMS (*μ*m)	4.06 (1.43)	3.32 (1.68)	0.042	4.04 (1.50)	3.14 (1.45)	0.022	3.84 (1.63)	3.01 (1.52)	0.048
	4.03 (6.14)	3.09 (8.39)		4.12 (5.99)	3.05 (7.17)		3.40 (5.75)	3.00 (7.64)	
Anterior LOA RMS (*μ*m)	15.30 (5.19)	12.49 (5.89)	0.014	15.09 (5.19)	11.88 (5.57)	0.019	14.49 (5.99)	11.34 (5.98)	0.048
	14.84 (22.26)	11.91 (27.33)		14.50 (21.64)	11.72 (25.47)		13.36 (22.82)	11.01 (27.02)	
Anterior *Z*_3_^−1^ (*μ*m)	−3.14 (1.68)	−2.51 (1.75)	0.075	−3.08 (1.67)	−2.48 (1.63)	0.125	−2.93 (1.78)	−2.36 (1.67)	0.239
	−3.38 (7.03)	−2.55 (8.82)		−3.16 (7.03)	−2.62 (8.31)		−2.63 (6.14)	−2.60 (8.60)	
Anterior *Z*_3_^1^Z_3_^1^ (*μ*m)	−0.07 (1.49)	0.08 (0.87)	0.397	−0.13 (1.57)	0.04 (0.82)	0.398	0.00 (1.51)	0.02 (0.83)	0.861
	−0.36 (5.38)	0.13 (3.96)		−0.42 (7.08)	0.13 (3.49)		−0.30 (5.32)	0.08 (3.36)	
Anterior *Z*_4_^0^ (*μ*m)	−0.86 (0.99)	−0.47 (0.92)	0.116	−0.88 (0.95)	−0.56 (0.84)	0.189	−0.85 (1.00)	−0.45 (0.87)	0.131
	−0.79 (4.34)	−0.28 (3.33)		−0.97 (3.74)	−0.49 (2.95)		−0.81 (3.94)	−0.16 (3.29)	
Posterior total RMS (*μ*m)	3.66 (1.19)	2.87 (1.32)	0.010	3.61 (1.33)	2.86 (1.16)	0.024	3.45 (1.52)	3.17 (2.75)	0.816
	3.48 (4.70)	2.83 (5.94)		3.09 (4.75)	2.79 (5.54)		2.94 (6.34)	2.60 (15.40)	
Posterior HOA RMS (*μ*m)	0.77 (0.48)	0.64 (0.34)	0.162	1.08 (0.37)	0.81 (0.32)	0.005	1.04 (0.45)	0.88 (0.67)	0.048
	0.76 (0.83)	0.47 (1.11)		0.94 (1.54)	0.79 (1.62)		0.89 (1.70)	0.73 (3.70)	
Posterior LOA RMS (*μ*m)	3.49 (1.15)	2.74 (1.27)	0.013	3.44 (1.28)	2.74 (1.12)	0.044	3.28 (1.46)	3.05 (2.67)	0.860
	3.38 (4.49)	2.66 (5.69)		2.97 (4.58)	2.68 (5.36)		2.83 (6.15)	2.47 (15.01)	
Posterior *Z*_3_^−1^ (*μ*m)	0.76 (0.41)	0.58 (0.37)	0.081	0.77 (0.43)	0.59 (0.37)	0.072	0.72 (0.46)	0.58 (0.37)	0.309
	0.74 (1.68)	0.57 (1.74)		0.71 (1.78)	0.61 (1.73)		0.64 (1.72)	0.65 (1.70)	
Posterior *Z*_3_^1^ (*μ*m)	0.02 (0.41)	−0.02 (0.24)	0.667	0.02 (0.44)	−0.01 (0.23)	0.554	−0.01 (0.40)	0.00 (0.23)	0.891
	0.09 (1.48)	0.00 (1.04)		0.05 (1.98)	−0.03 (0.95)		−0.01 (1.53)	−0.01 (1.00)	
Posterior *Z*_4_^0^ (*μ*m)	0.11 (0.22)	0.07 (0.23)	0.386	0.10 (0.20)	0.07 (0.19)	0.462	0.10 (0.23)	0.04 (0.17)	0.298
	0.11 (0.92)	0.03 (1.06)		0.12 (0.76)	0.07 (0.80)		0.11 (1.13)	0.04 (0.82)	

RMS: root mean square, HOA: higher order aberrations, LOA: lower order aberrations; *Z*_3_^−1^: vertical coma; *Z*_3_^1^: horizontal coma; *Z*_4_^0^: spherical aberration.

## Data Availability

The data used to support the findings of this study are available from the corresponding author upon request.
